# Transient Elevation of Liver Function Tests and Bilirubin Levels After Laparoscopic Cholecystectomy

**DOI:** 10.3390/medicina60111885

**Published:** 2024-11-17

**Authors:** Alexandros Giakoustidis, Menelaos Papakonstantinou, Christos Gkoutzios, Paraskevi Chatzikomnitsa, Areti Danai Gkaitatzi, Athanasia Myriskou, Petros Bangeas, Panagiotis Dimitrios Loufopoulos, Vasileios N. Papadopoulos, Dimitrios Giakoustidis

**Affiliations:** A’ Department of Surgery, General Hospital Papageorgiou, 56429 Thessaloniki, Greece; menelaospap.md@gmail.com (M.P.); christos.gkoutzios@gmail.com (C.G.); voula.hatzikomnitsa@gmail.com (P.C.); aretidanaegtz24@gmail.com (A.D.G.); myriskou@gmail.com (A.M.); pbangeas@gmail.com (P.B.); loufopoulosp@gmail.com (P.D.L.); papadvas@auth.gr (V.N.P.); dgiakoustidis@gmail.com (D.G.)

**Keywords:** laparoscopic cholecystectomy, liver function tests, bilirubin levels, postoperative complications

## Abstract

*Background and Objectives*: Laparoscopic cholecystectomy constitutes the current “gold standard” treatment of symptomatic gallstone disease. In order to avoid intraoperative vasculobiliary injuries, it is mandatory to establish the “critical view of safety”. In cases of poor identification of the cystic duct and artery leading to a missed intraoperative injury, patients present with elevated liver function tests (LFTs) or increased bilirubin postoperatively. The aim of this study is to present a series of patients of our institute with elevated liver enzymes and bilirubin after laparoscopic cholecystectomy in the absence of intraoperative injury or any other obvious etiology and to provide a possible explanation of this finding. *Materials and Methods*: From 2019 to 2023, 200 patients underwent elective laparoscopic cholecystectomy at the Papageorgiou General Hospital and at the European Interbalkan Medical Center of Thessaloniki utilizing the “critical view of safety” method. We retrospectively collected the intraoperative reports, and the pre- and postoperative imaging and laboratory studies of the patients included in this series. Postoperative LFTs and bilirubin levels were extracted and the reason for their transient elevation was examined. *Results*: From 200 cases of laparoscopic cholecystectomy, elevated LFTs and bilirubin were found in six patients on the first postoperative day, which is suggestive of a missed intraoperative injury. All patients were asymptomatic. During the investigatory workup, a triple-phase CT of the liver and/or an MRCP were ordered, but no pathological findings, such as biliary injury, hepatic artery injury or choledocholithiasis, were found. On postoperative day 3, LFTs and bilirubin levels decreased or normalized without any intervention. No postoperative complications were reported. *Conclusions*: In select cases, a transient increase in LFTs and/or bilirubin may be observed in the early postoperative period after elective laparoscopic cholecystectomy in the absence of an obvious etiology. A possible interpretation of these findings could involve the pneumoperitoneum or the anesthesia regimens used intra- or perioperatively. The specific cause, however, remains undetermined and yet to be examined by future studies.

## 1. Introduction

Acute cholecystitis is a common cause of hospital admission and is responsible for approximately 3 to 10% of all acute abdomen cases. Cholelithiasis is a major risk factor causing up to 95% of cholecystitis [1,2]. Gallstones are digestive fluid deposits formed in the gallbladder and may cause inflammation due to the occlusion of the cystic duct or impaired emptying of the gallbladder [3]. Globally, around 6% of the population have gallstones, with higher rates reported in females and in South America [1]. Gallstones may be asymptomatic at first, but approximately 20% of patients will develop complications at some point, including, but not limited to, cholecystitis, cholangitis or gallstone pancreatitis [4].

Laparoscopic cholecystectomy (LC) has essentially replaced the open technique since the 1990s and is currently the mainstay treatment for symptomatic gallstone disease [5]. Under general anesthesia, the abdomen is insufflated with carbon dioxide (pneumoperitoneum) at approximately 12–14 mmHg of intraabdominal pressure (IAP). After trocar placement, the triangle of Calot is dissected until the “critical view of safety” (CVS) is achieved based on three components (Strasberg’s criteria): (i) sufficient dissection in the hepatocystic triangle so that only two structures can be seen connected to the gallbladder (the cystic duct and cystic artery), (ii) separation of the lower one third of the gallbladder from the liver and exposure of the cystic plate and (iii) confirmation of the CVS by clearing all tissue from the hepatocystic triangle. The cystic artery and cystic duct are then clipped and divided, and the gallbladder is dissected from the liver bed and removed [5,6].

Many complications have been reported after LC, such as bile duct, vascular and even bowel injuries. Of them, biliary injuries represent the most significant ones, consisting of misidentifying the common bile duct or an aberrant right duct as the cystic duct and transecting or clipping them instead. This leads to bile leak, which in turn, if not managed during the operation, may have severe clinical manifestations (bile peritonitis). The lack of clear identification of the structures inside the hepatocystic triangle is among the most common causes of biliary injuries, which proves the significance of establishing CVS intraoperatively [7]. Vascular injuries during LC most commonly include injuries to the right hepatic artery, right hepatic vein or portal vein. They may occur in the presence of severe inflammation or anatomic variations, such as in the case of the caterpillar hump of the right hepatic artery, where the hepatic artery may be wrongly mistaken for the cystic artery. Transection of a vascular structure leads to bleeding during the operation, which may require conversion to open surgery. If a vascular injury remains obscure, it may present with hemodynamic instability in severe cases or with elevated liver function tests (LFTs) postoperatively in milder cases [8].

To avoid injury to biliary structures, intraoperative cholangiography may be performed in order to identify anatomic variations or gallstones in the biliary tree [9]. However, if a vasculobiliary injury is missed intraoperatively, multiple imaging modalities can be utilized to determine its type and location. Ultrasonography (US) and computed tomography (CT) could detect fluid collection in the perihepatic space. Of note, contrast-enhanced triple-phase liver CT is especially useful in detecting, for instance, active bleeding during the arterial phase, or portal vein thrombosis during the portal phase [10]. Magnetic resonance cholangiopancreatography (MRCP), on the other hand, is the imaging of choice to assess patients with suspected biliary tree injuries [11,12].

In certain cases, postoperative laboratory exams may indicate a vascular or biliary injury after LC without any clinical or imaging evidence. In our center we observed elevated LFTs and bilirubin levels in patients who underwent LC with successful CVS, with no intraoperative injuries and no evidence of injury in postoperative triple-phase CT or MRCP. The aim of this study is to address the clinical relevance and to discuss possible explanations of elevated LFTs and bilirubin levels after elective laparoscopic cholecystectomy in the absence of any intraoperative damage.

## 2. Materials and Methods

### 2.1. Study Design and Data Collection

This is a retrospective study of 200 patients who underwent elective laparoscopic cholecystectomy at the Papageorgiou General Hospital and the Interbalkan Medical Center of Thessaloniki, Greece from 2019 to 2023. The following data were extracted from the patients’ medical records in a predetermined data sheet: patient age, sex, BMI, preoperative laboratory tests, day of surgery, days of hospitalization, surgical technique, intraoperative complications, postoperative laboratory tests including WBCs, hemoglobin, SGOT, SGPT, total bilirubin and direct bilirubin, imaging studies including abdominal CT or MRI/MRCP, and patient vitals. Blood works were obtained on the day previous to the operation and on postoperative day 1. Patients were discharged on the first postoperative day unless alarming clinical or laboratory findings were present.

### 2.2. Surgical Technique

**Figure 1:**
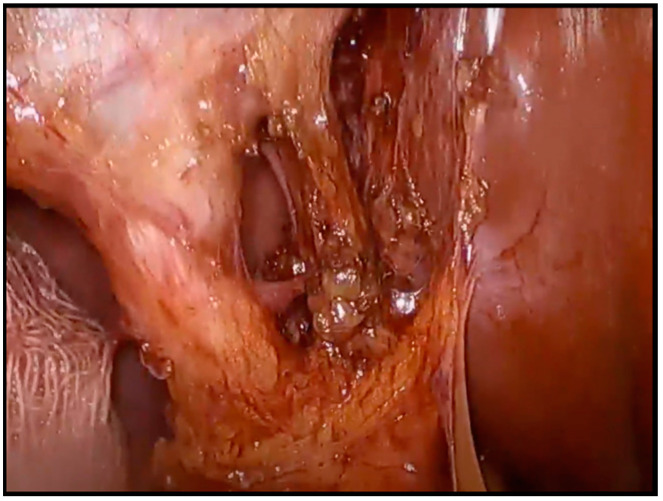
Intraoperative image of the “Critical View of Safety”. The cystic duct and cystic artery are recognized and then ligated in order to avoid injuries to vital structures.

Laparoscopic cholecystectomy was performed via the 4-port technique and Hasson pneumoperitoneum was established. All of the operations were performed by the same two surgeons (first surgeon and assistant). The “critical view of safety” was established in all the cases through careful dissection in the Calot’s Triangle before proceeding to the ligation of the cystic duct and artery (Figure 1). The gallbladder was removed through the umbilical port with a basket and a final laparoscopy was performed in order to exclude any potential damage to the involved structures. In case of uncertain identification of the Calot’s Triangle contents or of major complications including but not limited to major bleeding or bile duct injury, conversion to laparotomy was decided.

### 2.3. Inclusion and Exclusion Criteria

The inclusion criteria for this study were adult patients who underwent elective laparoscopic cholecystectomy for symptomatic gallstone disease from 2019 to 2023. Those with pre-existing liver diseases, clear-cut bile duct injuries, or any other extremity factors that may reflect in such findings as LFT and bilirubin levels were not involved in the analysis.

### 2.4. Data Analysis

Descriptive statistics were used to analyze our data. We focused on changes in LFTs and bilirubin levels from the day of surgery (day 0) to the postoperative day 3. Means and percentages were calculated to measure the amount of normalization, which is the most prominent feature in the present study.

Accessories were created in order to evaluate the relative increase and decrease in LFTs and bilirubin levels compared to the baseline for easier comprehension and interpretation of the results. The indices were calculated as follows:

Index Day 1 vs. Day 0 = (Day 1 value/Day 0 value) × 100%

Index Day 3 vs. Day 0 = (Day 3 value/Day 0 value) × 100%

## 3. Results

**Figure 2:**
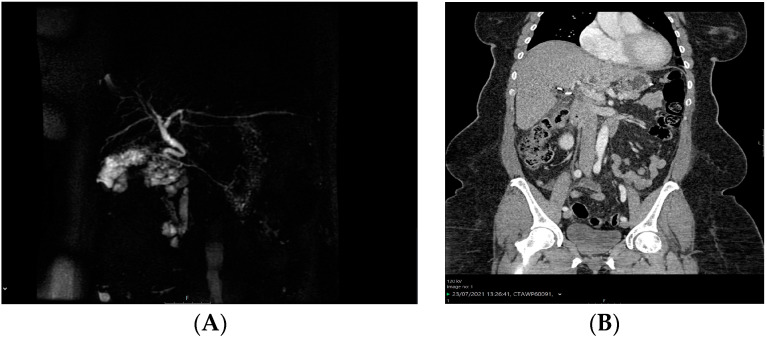
Imaging work-up on postoperative day 1 after the rise of the LFTs and bilirubin levels. (**A**) MRCP after laparoscopic cholecystectomy and (**B**) abdominal CT scan after laparoscopic cholecystectomy. No injuries or other intra-abdominal complications are present.

Two hundred (200) patients with symptomatic gallstone disease who underwent elective laparoscopic cholecystectomy from 2019 to 2023 were included in this study. Of them, an unusual elevation of both the liver function tests (LFTs) and bilirubin levels was observed in six patients (3%) on the first postoperative day and intraoperative damage to vital structures was suspected. Patient demographics and associated comorbidities are shown in Table 1. The patients remained asymptomatic, however a work-up was initialized in order to safely exclude any possible overseen adverse events. Since clinical examination was normal, imaging tests were ordered for all six patients including a triple-phase CT and an MRCP. The MRCP of patient 2 and the CT scan of patient 6 are shown in Figure 2. Neither the triple-phase CT nor the MRCP scans revealed any pathological findings, such as injuries to the hepatic artery, bile duct injuries, or remaining gallstones. Since the imaging examinations of the other 4 patients were similar, they were not reported herein. An intraoperative injury was therefore safely excluded; however, the hospitalization was prolonged in order to monitor the patients with serial laboratory tests.

Blood tests were obtained on postoperative day 1 and day 3 and the biochemistry laboratory values including the LFTs (SGOT and SGPT) and the bilirubin (total and direct) levels for each patient are shown in Table 2. Compared to the preoperative counterparts, a substantial elevation of both the bilirubin and the LFTs was present on postoperative day 1. More specifically, the mean values of SGOT and SGPT were increased by 161.9% and 182.7%, respectively, while the mean values of total and direct bilirubin increased by 160% and 158.8%, respectively (Table 3). On postoperative day 3, the laboratory values started to drop or returned to normal without any intervention and all six patients were discharged.

## 4. Discussion

The present study shows that a transient elevation of LFTs and bilirubin levels may be observed in certain patients after elective laparoscopic cholecystectomy in the absence of any intraoperative damage. Those abnormal laboratory values returned to normal within two days without any intervention, while the patients remained entirely asymptomatic. However, that finding led to an imaging work-up that was stressful both for the patient and for the surgeon as well increasing the cost of the hospitalization. The length of stay was also increased compared to the usual discharge of patients on postoperative day 1 at our institution. In order to avoid the above circumstances in the future, the reason for that transient elevation should be further investigated. In some of our patients, comorbidities were present, such as diabetes mellitus and hypertension, which may have impacted the liver function after surgery. The pneumoperitoneum could also be the cause of that transient elevation of the LFTs and bilirubin, a finding that has been reported by Tan et al. after both laparoscopic cholecystectomy and laparoscopic colorectal cancer resection [13]. Other causes could involve the intraoperative maneuvering of the liver or the administration of certain anesthesiology or other perioperative drugs, but larger systematic studies are needed to confirm any correlation. Even the dissection method was investigated, but no relationship was found between dissector type and changes in the postoperative LFTs [14]. However, the drop of the elevated enzymes on the third postoperative day without any intervention could also support the self-limited incidental and benign nature of cholestasis after laparoscopic surgery.

To our knowledge, the first report of a transient LFTs elevation after laparoscopic cholecystectomy was mentioned in 1994 by Halevy et al. who found a significant increase in liver enzymes without the presence of intraoperative damage [15], while the reason for that elevation was argued to be the increased intra-abdominal pressure due to pneumoperitoneum [16]. Similar findings have also been reported by other studies in which the abnormal findings were rendered to liver ischemia due to pneumoperitoneum pressure and other confounding factors, such as the patient’s BMI, the duration of surgery or specific anesthetic agents [13,17,18,19,20]. Of note, a Randomized Controlled Trial by Neogi et al. compared the effect of normal to that of low-pressure pneumoperitoneum in the postoperative levels of liver enzymes. They found that a normal pressure pneumoperitoneum was associated with a significant increase of both SGOT and SGPT 24 h after the operation [21]. The liver enzyme abnormalities were found to be the result of a decreased blood flow to the liver due to the pneumoperitoneum pressure [22]. Accordingly, Gupta et al. reported less postoperative abnormalities in LFTs in patients undergoing laparoscopic cholecystectomy with low-pressure pneumoperitoneum [18]. However, there are conflicting data in the literature since Bickel et al. concluded that the induction of pneumoperitoneum did not affect the postoperative liver enzyme levels. In their study of 1034 patients who underwent elective laparoscopic cholecystectomy, only 41 manifested mild LFTs increase while choledocholithiasis was present in 9 of them. All of the abnormal laboratory values reported and the associated liver malfunction were clinically insignificant since no patients developed any symptoms. However, this might be different in patients with an underlying liver disease; therefore, care should be taken to avoid unnecessary burdens on corresponding cases [23,24].

Furthermore, the immune response to the postoperative stress may result in local inflammation and damage to the liver cells, leading to elevated SGOT and SGPT serum levels [25]. Moreover, surgical site fluid accumulation could affect the bile flow, thus increasing the bilirubin levels. Ligation or banding of the cystic duct could cause interruption or edema that hinders the biliary flow. Buildup of bile ensues and subsequently bilirubin levels increase [6]. Thus, the transient elevation of bilirubin levels seen in the first days after laparoscopic cholecystectomy could possibly result from bile reabsorption. Eventually, the obstruction is resolved, and bilirubin levels return to normal. Sometimes, low bile flow develops spontaneously during surgery, due to surgical instrument manipulation again causing bile retention and elevation of bilirubin levels after laparoscopic cholecystectomy. The surgical and psychosomatic stress related to the laparoscopic cholecystectomy may lead to the release of inflammatory mediators and cytokines [26]. Among other effects, those mediators have the potential to trigger transient hepatocellular injury and impaired bile excretion, which could consequently lead to the transient elevation of LFTs and bilirubin levels observed postoperatively.

Even though at our institution we routinely test the postoperative biochemistry labs in all patients after laparoscopic cholecystectomy, other studies conclude that measuring the LFTs after elective laparoscopic cholecystectomy should be performed only when clinical indications are present [27,28]. That is because changes in LFTs do not seem to predict complications [29]. Al Jaberi et al. declare that in the absence of clinical findings, direct bilirubin and alkaline phosphatase assessment is preferred to evaluate those patients postoperatively, since no significant difference between groups of increased and normal LFTs was present regarding patient age or the duration of the surgery [28]. Saber et al. also observed elevated LFTs after laparoscopic cholecystectomy in the absence of intraoperative injury that normalized after 7 days without any clinical symptoms. The rise of the enzymes was attributed to the high pneumoperitoneum pressure; however, they highlighted the self-limited nature of this pattern in order to avoid unnecessary workup and cost in the future [30,31].

### 4.1. Clinical Implications

A significant point in this study is that the sudden LFT and bilirubin elevations resolved themselves naturally without any intervention, after which the patients were discharged asymptomatic without any other postoperative complications. This implies that these abnormalities are benign and self-limiting but should be expected in a small percentage of laparoscopic cholecystectomies.

Nevertheless, in select cases, proper interpretation of these insights is mandatory and all possible factors that can invalidate these explanations, especially medical conditions, must be considered. Therefore, a persistent rise in LFTs and bilirubin levels may require careful diagnosis and investigation for other causes, such as bile duct injuries, hepatic dysfunction, or complications during and after the operation. Finally, in patients with a coexisting liver dysfunction, laparoscopic cholecystectomy with a lower pressure pneumoperitoneum should be considered in order to avoid further liver impairment.

### 4.2. Limitations

This study has several limitations that should be acknowledged. The retrospective design of the study makes the admission of the unforeseen and undeterminable confounding factors inevitable. The fact that the study consisted of six patients restricted the extent to which the results could be generalized. With a small sample size, the chance of sampling error increases, making it impossible to assess the association or variance. Additionally, many confounding variables may interfere with the results, such as the patient’s physical performance status, the BMI, smoking habits, the duration of surgery, or any underlying medical conditions. Moreover, no long-term follow-up data beyond postoperative day 3 were available that may have provided some extra insights into the long-term results and the possible complications. Finally, our findings do not apply in cases of an emergent cholecystectomy, as an ongoing inflammation interferes with the pathophysiology and may distort the clinical picture of the patient.

### 4.3. Future Direction

The present study offers a possible basis for future investigation directed at scouring the intricate mechanisms and clinical meaning of the transient elevation of LFTs and bilirubin levels following laparoscopic cholecystectomy. Potential research directions include the following:

Prospective Studies: A further step to getting consistent and reliable data would be to enlarge the scale of an ongoing study, which entails the inclusion of a larger sample size and clearly defined inclusion and exclusion criteria. Prospective studies are likely to minimize the effects of confounding factors, and standardize data collection and methodologies, which will reduce bias. Such studies may lay the foundation for identifying the real prevalence, progression, and risk factors linked with continuously changing liver enzymes and bilirubin levels.

Mechanistic Studies: Investigating the possible mechanisms responsible for transient elevation of LFTs and bilirubin is the critical foundation for further study in order to present a comprehensive approach to the subject. Investigations examining inflammatory factors, physiology of bile ducts or hepatocellular injury are the main points of interest.

Risk Factor Analysis: Identification of such risk factors, like patient-specific characteristics (age, gender, comorbidities), surgical techniques (length of surgery, approach), or perioperative care (drug use, anesthetic technique) that could predispose individuals to short-lived abnormal laboratory tests might lead to the creation of preventive strategies or methods targeted at specific subgroups.

Long-term follow-up: Long-term, large-scale follow-up studies might demonstrate therapeutic differences of prolonged cytokine peaks versus their transient accumulation in the development of liver diseases and other types of biliary or other long-term sequelae. Such investigations would give a clue to the disease progress and phenotype of individuals experiencing transient spikes, presenting evidence for thoughtful decision-making and standardized protocols for patient follow-up.

## 5. Conclusions

The present study shows that a transient increase of LFTs and bilirubin after elective laparoscopic cholecystectomy without evidence of any intraoperative adverse events is self-limited within a couple of days. However, the specific mechanisms remain largely unknown and should be further investigated in the future. With the utilization of laparoscopic cholecystectomy as the “gold standard” treatment for symptomatic gallstone disease, it is paramount that healthcare workers be aware of such a phenomenon and its clinical implications. Effective collaboration among surgeons, anesthesiologists, nurses and other healthcare professionals can contribute to managing and improving patient outcomes after laparoscopic surgery. Hopefully, our study elevates an area for further research and also calls for ongoing investigation for the human physiological response to laparoscopic intervention.

## Figures and Tables

**Table 1:** Patient demographics and preoperative characteristics.

Patient	Age	Gender	BMI (kg/m²)	ASA	Comorbidities
1	35	M	23.2	I	None
2	62	F	25.1	I	None
3	38	F	24.9	II	Hypertension
4	41	F	21.8	I	None
5	42	M	28.3	II	Hypertension, DM, dyslipidemia
6	59	F	31	II	Hypertension, DM

BMI, body mass index; ASA, American Society of Anesthesiologists; DM, Diabetes Mellitus.

**Table 2:** Preoperative and postoperative LFTs and bilirubin laboratory values.

Patient	SGOT			SGPT			Total Bilirubin			Direct Bilirubin		
	Day 0	Day 1	Day 3	Day 0	Day 1	Day 3	Day 0	Day 1	Day 3	Day 0	Day 1	Day 3
1	93	138	81	75	137	142	2.0	3.2	1.2	1.7	2.7	0.6
2	65	111	64	71	125	95	1.8	1.9	1.0	1.5	1.5	0.6
3	71	143	69	94	151	140	1.9	2.7	1.1	1.4	1.8	0.7
4	111	247	98	83	212	91	1.6	2.2	1.2	1.0	1.7	0.5
5	221	378	197	110	260	173	1.1	1.6	0.8	0.4	1.0	0.3
6	169	291	134	118	256	192	1.6	2.2	1.0	1.1	1.6	0.5

**Table 3:** Relative changes between preoperative and postoperative LFTs and bilirubin levels.

Index	SGOT	SGPT	Total Bilirubin	Direct Bilirubin
Index Day 1 vs. Day 0 (%)	161.9	182.7	160.0	158.8
Index Day 3 vs. Day 0 (%)	65.9	189.3	60.0	35.3

## Data Availability

The raw data supporting the conclusions of this article will be made available by the authors on request.
